# Cardiac Complications Following Cardiac Surgery Procedures

**DOI:** 10.3390/jcm9103347

**Published:** 2020-10-18

**Authors:** Jakub Udzik, Sandra Sienkiewicz, Andrzej Biskupski, Aleksandra Szylińska, Zuzanna Kowalska, Patrick Biskupski

**Affiliations:** 1 Department of Cardiac Surgery, Pomeranian Medical University, Powstańców Wielkopolskich 72, 70-111 Szczecin, Poland; sasi@onet.com.pl (S.S.); a.biskupski@vp.pl (A.B.); 2 Department of Medical Rehabilitation and Clinical Physiotherapy, Pomeranian Medical University in Szczecin, Żolnierska 54, 71-210 Szczecin, Poland; aleksandra.szylinska@gmail.com; 3 Department of Mathematics and Informatics, Adam Mickiewicz University, Uniwersytetu Poznańskiego 4, 61-712 Poznań, Poland; zuzannakowalska7@o2.pl; 4 Lincoln Medical Center, 234E 149th St, Bronx, New York, NY 10451, USA; patrickbiskupski@gmail.com

**Keywords:** cardiac surgery, postoperative complications, bradycardia

## Abstract

Background: Elderly patients and those with multiple concomitant disorders are nowadays qualified for cardiac surgery procedures, which is related to higher incidence of the postoperative complications. Aim: The aim of this study was a retrospective analysis of the perioperative factors potentially contributing to occurrence of cardiac incidents after cardiac surgery procedures. Methods: Data of 552 patients of the cardiac surgery clinic were collected and analyzed. Data concerning medical history, previous treatment, laboratory results, additional tests results, operation and hospitalization period were examined. Results: In the study population of 552 patients, cardiac complications were observed in 49.5% of them. Among cardiac complications, the most frequent were supraventricular tachycardia (30.1%) and atrial fibrillation (27.4%). Postoperative bradycardia occurred in 5.25% patients, half of whom required temporary cardiac pacing. Conclusions: The risk of incidence of cardiac complications after cardiac surgery procedures depends mostly on patient’s age, EuroSCORE Logistic (ESL) score, left ventricular ejection fraction, myocardial hypertrophy, presence of paroxysmal AF and coincidence of nephrological complications. The necessity of performing more than one heart defibrillation after removing aortic cross-clamp favors early postoperative bradycardia. Considering the outcomes of this study, continuing reperfusion at least until 1/3 of the aortic cross-clamp time brings no additional benefits to the patients.

## 1. Introduction

One of the areas of cardiac surgery in which great progress was achieved is broadening the group of patients qualified for these procedures. Elderly patients and those with multiple concomitant disorders [1] are nowadays qualified for these procedures. Higher perioperative risk in these patients is related to higher incidence of the postoperative complications, which are particularly dangerous in this group of patients [2]. In all operative specialties, postoperative complications concern wide range of patients [3] and are a substantial problem. These complications may involve various types of disorders, depending on the patient and his/her clinical condition. This study focuses on cardiac incidents following cardiac procedures including:

- Supraventricular tachycardia (SVT);
- Atrial Fibrillation (AF);
- Bradycardia;
- Atrioventricular block (AVB);
- Perioperative myocardial infarction (MI);
- Cardiac arrest (CA);
- Death due to cardiac causes—cardiac death (CD).

Numerous studies aimed at identifying factors affecting postoperative complications after cardiac surgery procedures have been published [4,5,6]. Equally, many studies were dedicated to finding novel solutions to prevent these complications [7,8,9]. Nonetheless, the exact causes of complications, such as atrial fibrillation or bradycardia and processes like spontaneous return of heart rhythm after removing aortic cross-clamp are still elusive.

The authors in this study attempted to provide new information concerning these issues throughout the analysis of the patient’s preoperative condition, extracorporeal circulation parameters, course of the surgery, tests results and postoperative period.

The aim of this study was a retrospective analysis of the perioperative factors potentially contributing to occurrence of cardiac incidents after cardiac surgery procedures.

## 2. Experimental Section

Data of 552 patients of the cardiac surgery clinic were collected. Patients included in this study underwent cardiac surgery procedures between January and July 2018. In the study population, there were 157 women (28.4%) and 395 men (71.6%) with a mean age of 65.7 ± 8.66 years.

Patients qualified for all kinds of cardiac surgery procedures with the use of cardiopulmonary by-pass (CPB), both planned and emergency surgeries were included in this study. Data concerning medical history, previous treatment, laboratory results, additional tests results, operation and hospitalization period were examined. Additional attention was given to postoperative complications and measures applied to contain them.

Seven crucial cardiac complications were determined and their occurrence was the endpoint of this study: supraventricular tachycardia (SVT), atrial fibrillation (AF), bradycardia, atrioventricular block (AVB), myocardial infarction (MI), cardiac arrest (CA) and death due to cardiac causes—cardiac death (CD).

SVT was defined as heart rate above 100 beats per minute with narrow QRS in ECG monitoring (QRS duration time <120 ms), including sinus tachycardia, atrial fibrillation, atrial flutter, atrioventricular nodal reentrant tachycardia (AVNRT) and atrioventricular reentrant tachycardia (AVRT).

Postoperative myocardial infarction was defined as 5-fold increase in CK-MB level, comparing to preoperative value, with ECG changes specific for myocardial infarction.

Cardiac death was defined as decease caused by primary circulatory failure or sudden cardiac arrest which occurred in the absence of other (non-circulatory) predisposing factors, such as respiratory failure, acute renal failure or shock.

The reperfusion strategy protocol used in our Clinic includes maintaining reperfusion until such time when hemodynamically sufficient heart rhythm is restored. Obtaining normal sinus rhythm is not obligatory, SVT or AF is also acceptable if generates normal arterial blood pressure.

Patients were divided into several groups basing on the analyzed factor. Dichotomous analysis was used in order to obtain some of those groups. Most common complications’ sets are presented in Table 1. The other groups were obtained considering some of the key issues related to cardiac surgeries, such as decrease of hematocrit level after the initiation of cardiopulmonary by-pass, necessity of defibrillating the heart after removing the aortic cross-clamp and the adequate reperfusion time for each patient.

Criteria applied to enroll patients into consecutive study groups:

- occurrence of any of the cardiac complications
  (study group: patients with any cardiac complications, *n* = 279;
  control group: patients without cardiac complications, *n* = 273)
- reperfusion time shorter than 1/3 of the aortic cross-clamp time
  (study group: patients with reperfusion time shorter than 1/3 of the aortic cross-clamp time, *n* = 200;
  control group: patients with reperfusion time at least 1/3 of the aortic cross-clamp time, *n* = 352)
- necessity of performing more than one defibrillation of the heart after removing the aortic cross-clamp
  (study group: patients who required more than one defibrillation after removing the aortic cross-clamp, *n* = 116;
  control group: patients who required not more than one defibrillation after removing the aortic cross-clamp, *n* = 436)
- decrease in hematocrit level after the initiation of cardiopulmonary by-pass greater than 30% of the initial value
  (study group: patients with decrease in hematocrit of at least 30%, *n* = 79;
  control group: patients with decrease in hematocrit smaller than 30%, *n* = 473)
- occurrence of postoperative bradycardia
  (study group: patients in whom occurred bradycardia, *n* = 21;
  control group: patients in whom bradycardia did not occur, *n* = 531)
- occurrence of isolated postoperative atrial fibrillation
  (study group: patients in whom occurred isolated postoperative atrial fibrillation, *n* = 75;
  control group: patients without cardiac complications, *n* = 273)
- occurrence of isolated supraventricular tachycardia
  (study group: patients in whom occurred isolated supraventricular tachycardia, *n* = 88;
  control group: patients without cardiac complications, *n* = 273)
- occurrence of postoperative atrial fibrillation combined with supraventricular tachycardia
  (study group: patients in whom occurred postoperative atrial fibrillation combined with supraventricular tachycardia, *n* = 61;
  control group: patients without cardiac complications, *n* = 273)

To increase the credibility of obtained results the study groups of patients with isolated complications were compared to the control group of patients with no cardiac complications. The exception was the study group of patients with postoperative bradycardia as in none of the patients was it an isolated complication. Regarding the above, that group of patients was compared to the rest of the study population.

The analyzed perioperative factors are demonstrated in Table 2.

Statistical analysis was conducted using licenced Statistica 12.0 software (StatSoft, Inc., Tulsa, OK, USA). Descriptive statistics was used in order to characterize the study groups: mainly, averages, standard deviations and medians. Evaluation of the normality of distribution of the analyzed variables was conducted using Shapiro–Wilk test. Homogeneity of variance was assessed using Levene’s test. Normal distribution of analyzed variables was not obtained. For that reason, the U Mann–Whitney test was conducted to assess these variables. Qualitative data were analyzed using eighter chi2 test or chi2 test with Yates correction, depending on their quantity. Significance level *p* ≤ 0.05 was assumed.

Multiple linear regressions were used for the analysis of the data potentially contributing to spontaneous circulation return.

## 3. Results

In the study population of 552 patients, cardiac complications were observed in 273 of them (49.5%). Among cardiac complications, the most frequent were supraventricular tachycardia (30.1%) and atrial fibrillation (27.4%). The exact results are presented in Table 3.

Perioperative parameters (Table 2), which values significantly differed (*p* < 0.05) in given study and control groups were described below. Other parameters did not show significant differences.

The analysis of factors predisposing to the occurrence of postoperative cardiac complications revealed the contribution of features depicted in Table 4.

Factors predisposing to isolated SVT occurrence (88 patients—15.9%) are demonstrated in Table 5.

Contributors to increased risk of isolated AF (75 patients—13.6%) are depicted in Table 6.

Occurrence of AF with concomitant SVT (61 patients—11.1%) is related to factors demonstrated in Table 7.

Postoperative bradycardia occurred in 29 patients (5.3%) and was associated with factors demonstrated in Table 8.

In the group of 29 patients suffering from postoperative bradycardia, there were 10 who underwent procedures involving aortic valve replacement (AVR), which accounts for 34.5% (vs. 23.9% in the study population). In this group, there are 11 patients who required more than one heart defibrillation after removing aortic cross-clamp and 18 patients who required one or no defibrillation. The percentage of patients who underwent AVR in both these groups is 36.4% and 33.3%, respectively, and the two-proportion test showed no significant difference between the groups (significance level *p* < 0.05 was assumed). A total of 15 patients suffering from bradycardia required temporary cardiac pacing (using epicardial or endocavitary electrode), although none of them required pacemaker implantation.

Atrioventricular block (AVB) occurred in 13 patients. In 6 patients, it was a 3rd degree AVB (total AVB); 5 of these patients required temporary cardiac pacing (using epicardial or endocavitary electrode). None of the patients required pacemaker implantation during hospitalization at the cardiac surgery department (normal sinus rhythm returned or efficient replacement rhythm was obtained). A total of 3 of the patients who suffered from total AVB underwent double valvular procedure regarding mitral and tricuspid valve, the other 3 patients underwent AVR procedure, AVR combined with ascending aorta prosthesis implantation and coronary artery by-pass grafting (CABG) combined with AVR.

Postoperative myocardial infarction occurred in 5 patients (0.9%), 3 of whom underwent CABG, 1 patient underwent valvular procedure and 1 underwent complex procedure. By-pass revision was necessary in 2 patients; another 2 required PTCA procedure. In one patient, the procedure was palliative and revascularization of the narrowed vessels was not possible.

The outcomes of patients in whom the reperfusion time was shorter than 1/3 of the aortic cross-clamp time (study group) were compared to the outcomes of patients in whom this time was at least 1/3 (control group). The only factor that demonstrated a significant difference between the groups was pressor amines administration, which was more often required in the study group (67 patients—33.5% vs. 77 patients—21.9% in the control group, *p* = 0.003).

The study group included patients with higher ESL score (7.95 ± 8.35 vs. 6.38 ± 7.16, *p* < 0.001), longer perfusion time (82.72 ± 26.81 min vs. 58.71 ± 22.73 min in the control group, *p* < 0.001), longer aortic cross-clamp time (64.04 ± 21.37 min vs. 34.88 ± 14.05 min in the control group, *p* < 0.001) and patients who more frequently underwent valvular and complex procedures (160 patients—80% vs. 57—16.2% in the control group, *p* < 0.001).

A total of 116 patients (21%) required more than one heart defibrillation in order to restore normal sinus rhythm. They were patients with lower BMI (28.28 ± 4.41 vs. 29.24 ± 4.47 in the control group, *p* = 0.021), longer perfusion time (73.75 ± 33.69 min vs. 65.72 ± 24.51 min in the control group, *p* = 0.048) and patients who more frequently underwent complex procedures (59 patients—50.9% who underwent CABG in the study group vs. 276 patients—63.3% who underwent CABG in the control group, *p* = 0.015).

Patients in whom occurred spontaneous return of heart rhythm after removing aortic cross-clamp had lower calcium level in the last measurement during CPB (1.188 ± 0.075 mmol/L vs. 1.205 ± 0.093 mmol/L in the study group, *p* = 0.019) than patients who required heart defibrillation. Binary logistic regression of parameters potentially contributing to the spontaneous return of heart rhythm after removing aortic cross-clamp revealed an important role of patient’s calcium level in this process (*p* = 0.03). Nevertheless, the optimal calcium level predisposing to spontaneous return of heart rhythm could not be determined, as the applied mathematical models failed to provide unequivocal results. The Mann–Whitney–Wilcoxon test result confirms that the occurrence of spontaneous return of heart rhythm after removing aortic cross-clamp differs depending on calcium level.

Postoperative atrial fibrillation occurred in 151 patients (27.4% of the study population), among whom 67.5% were male. This complication’s incidence was highest in the first day after surgery (34.2%), then in the second day (23.1%) and the third (19.7%). The latest onset of this complication among the patients included in this study took place on the fourteenth day of hospitalization. The preferred method of treatment was pharmacological cardioversion with the use of amiodarone. Electrical cardioversion was necessary in 6.6% of the patients, 60% of whom were patients with concomitant SVT.

The decrease of hematocrit level after initiation of CPB greater or equal to 30% of the initial value is not connected with higher frequency of cardiac complications.

Cardiac arrest occurred in 15 patients (3 women and 12 men), which accounts for 2.7% of the study population. The most common mechanism of cardiac arrest was ventricular fibrillation—VF (30%), followed by asystole (26.7%), pulseless electrical activity—PEA (20%) and pulseless ventricular tachycardia—pVT (6.7%).

In two patients, the primary mechanism of cardiac arrest were not determined. The resuscitation was successful in 9 patients (60%).

A total of 17 patients died (6 women and 11 men) and the total mortality in the study population was 3.1%. In 4 patients, the cause of death was multiple organ failure. Cardiac death (CD) occurred in 13 patients (6 women and 7 men). The average age of the deceased was 66.62 ± 8.78 years and the average ESL score was 23.7 ± 7.55. Complex procedures were performed in 9 patients, CABG in 3 patients and valvular procedure in 1 patient. A total of 61.5% of patients in this group were diagnosed with arterial hypertension, 38.5% with diabetes and 69.2% had at least one previous myocardial infarction.

## 4. Discussion

The aim for this study was to analyze the perioperative factors and their influence on the incidence of cardiac complications after cardiac surgery procedures. The obtained results clearly depict factors which predispose patient to having certain complications or their constellation. The most frequent complications were supraventricular tachycardia, atrial fibrillation and supraventricular tachycardia combined with atrial fibrillation. Sinus tachycardia following cardiac surgery procedures may be a physiological response of the circulatory system, but it may also be a sign of pathology such as inadequate analgesia, insufficient fluids administration or ion imbalance. For the needs of this study the authors assumed that occurrence of supraventricular tachycardia in patients after cardiac surgery is primarily not the direct result of the procedure, but is a reaction of the organism to other disturbances, which analysis lies beyond the frames of this study. For this reason, SVT is counted among postoperative complications. The authors believe that noticing this irregularity is an important premise to look for its cause.

Particularly relevant is the association between the number of defibrillations and incidence of postoperative bradycardia demonstrated in this study. Authors of this article were unable to reach any similar reports in the literature. Incidence of bradycardia was so far reported only in patients with implanted ICD devices [10,11] and also in patients after successful reanimation [12]. Nevertheless, in none of these reports is bradycardia linked with heart defibrillation. Direct heart defibrillation (power of 10–30 J) was performed in patients included in this study during CPB. In some of those patients, bradycardia appeared after up to 24 h after the procedure, without any other perceptible reasons. This suggests that the necessity of heart defibrillation after removing aortic cross-clamp is a risk factor for early postoperative bradycardia, even if satisfactory heart rate was primarily obtained. Statistical analysis proved that the number of patients who underwent AVR procedure in the postoperative bradycardia group does not undermine the conclusion about the relationship between heart defibrillation and occurrence of bradycardia.

Of no small significance is also the outcome of comparison of the results of patients with different reperfusion time (≥1/3 aortic cross-clamp time vs. <1/3). It proves the rule of continuing reperfusion at least until 1/3 of the aortic cross-clamp time [13] does not provide better treatment results. Patients in the study group were significantly more frequently administered catecholamines infusions; however, due to the retrospective nature of this study, only the necessity for drug administration was noted, not the dosage. Patients in the study group had significantly longer perfusion and aortic cross-clamp time, with a higher percentage of valvular and complex procedures in this group. Considering the above, it is clear that continuing reperfusion at least until 1/3 of the aortic cross-clamp time brings no additional benefits to the patients.

The studies conducted so far in this area were mainly focused on specific complications [14,15]. The advantage of this study is taking under consideration some range of complications, which frequently coexist and potentially influence one another.

## 5. Conclusions

The risk of incidence of cardiac complications after cardiac surgery procedures depends mostly on patient’s age, ESL score, left ventricular ejection fraction, myocardial hypertrophy, presence of paroxysmal AF and coincidence of nephrological complications. The necessity of performing more than one heart defibrillation after removing aortic cross-clamp favors early postoperative bradycardia. Considering the outcomes of this study, continuing reperfusion at least until 1/3 of the aortic cross-clamp time brings no additional benefits to the patients.

## Figures and Tables

**Table 1:** Results of the dichotomous analysis of cardiac complications.

Complication Sets	Patients (*n*)	%
No complications	273	49.46
SVT (isolated)	86	15.58
AF (isolated)	75	13.59
SVT and AF	61	11.05

Legend: AF—atrial fibrillation, SVT—supraventricular tachycardia.

**Table 2:** Analyzed perioperative factors.

Preoperative	Intraoperative	Postoperative
age sex ESL BMI initial laboratory results (CK-MB, CRP, Hb, K^+^, Hb_A1C_) LV ejection fraction LA and LV diameter CCS and NYHA scores arterial hypertension AF (paroxysmal and persistent) diabetes dyslipidemia myocardial infarction in the past coronary vessels angioplasty cardiac arrest implanted heart stimulator endocarditis	surgery type myocardial protection protocol spontaneous return of heart rhythm after removing aortic cross-clamp number of defibrillations required to restore normal heart rhythm maximal defibrillation power fluid balance from the operating room perfusion time aortic cross-clamp time reperfusion time time of ischemia CPB parameters (Hb, pH, pCO_2_, pO_2_, K^+^, Ca^2+^) intraoperative levozymendan infusion	atrial fibrillation myocardial infarction cardiac arrest catecholamines infusion postoperative levozymendan infusion temporary epicardial stimulation atrioventricular block bradycardia reoperation nephrological complications renal replacement therapy stroke postcardiotomy delirium death red blood cells transfusion peak values of laboratory results(CK-MB, CRP)

Legend: AF—atrial fibrillation, CCS—Canadian Cardiovascular Society angina grade, CK-MB—creatine kinase-MB, CPB—cardiopulmonary by-pass, CRP—C-reactive protein, ESL—EuroSCORE Logistic, Hb—hemoglobin, Hb_A1C_—glycated hemoglobin, LA—left atrium, LV—left ventricle, NYHA—New York Heart Association score.

**Table 3:** Prevalence of cardiac complications in the study population.

Complications	Patients (*n*)	%
Supraventricular tachycardia	166	30.1
Atrial fibrillation	151	27.4
Bradycardia	29	5.3
Cardiac arrest	15	2.7
Cardiac death	13	2.4
Atrioventricular block	13	2.4
Myocardial infarction	5	0.9

**Table 4:** Factors predisposing to the occurrence of postoperative cardiac complications.

	Study Group	Control Group	*p*-Value
Age (years ± SD)	66.47 ± 8.8	64.93 ± 8.45	0.014
ESL score (± SD)	7.59 ± 7.66	6.34 ± 7.59	0.004
LV ejection fraction (% ± SD)	45.78 ± 12.3	48.53 ± 10.3	0.023
LA dilatation (cm ± SD)	4.26 ± 0.67	4.14 ± 0.69	0.02
LV dilatation (cm ± SD)	5.34 ± 0.83	5.19 ± 0.77	0.037
Paroxysmal AF *n* (%)	29 (10.5)	12 (4.4)	0.006
Nephrological complications *n* (%)	91 (33)	61 (22.1)	0.004

Legend: AF—atrial fibrillation, ESL—EuroSCORE Logistic, LA—left atrium, LV—left ventricle.

**Table 5:** Factors predisposing to the occurrence of isolated SVT.

	Study Group	Control Group	*p*-Value
Male sex *n* (%)	72 (82)	192 (70)	0.025
Infective endocarditis in medical history *n* (%)	6 (7)	5 (2)	0.042

**Table 6:** Factors predisposing to the occurrence of isolated AF.

	Study Group	Control Group	*p*-Value
Age (years ± SD)	68.72 ± 8.5	64.93 ± 8.45	0.001
Paroxysmal AF *n* (%)	11 (15)	12 (4)	0.003

Legend: AF—atrial fibrillation.

**Table 7:** Factors predisposing to the occurrence of AF with concomitant SVT.

	Study Group	Control Group	*p*-Value
Age (years ± SD)	69.28 ± 7.12	64.93 ± 8.45	<0.001
ESL score (± SD)	8.02 ± 7.39	6.34 ± 7.59	0.006
Paroxysmal AF *n* (%)	10 (16.4)	12 (4.4)	0.002
Nephrological complications *n* (%)	23 (37.7)	61 (22.1)	0.011

Legend: AF—atrial fibrillation, ESL—EuroSCORE Logistic, LA—left atrium, LV—left ventricle.

**Table 8:** Factors predisposing to the occurrence of postoperative bradycardia.

	Study Group	Control Group	*p*-Value
Age (years ± SD)	68.41 ± 6.15	65.55 ± 8.75	0.039
ESL score (± SD)	9.96 ± 9.35	6.78 ± 7.52	0.004
LA dilatation (cm ± SD)	4.43 ± 0.59	4.19 ± 0.68	0.028
Perfusion time (min ± SD)	86.55 ± 38	66.34 ± 185	0.003
Aortic cross-clamp time (min ± SD)	55 ± 30	44.19 ± 152	0.042
Necessity of performing more than one heart defibrillation after removing aortic cross-clamp *n* (%)	11 (9.5)	18 (4.1)	0.022

Legend: ESL—EuroSCORE Logistic, LA—left atrium.
